# Whole genome sequencing and antimicrobial resistance among clinical isolates of *Shigella sonnei* in Addis Ababa, Ethiopia

**DOI:** 10.1371/journal.pone.0313310

**Published:** 2024-11-12

**Authors:** Basha Ayele, Adane Mihret, Zeleke Mekonnen, Tesfaye Sisay Tessema, Kalkidan Melaku, Maeruf Fetu Nassir, Abaysew Ayele, Dawit Hailu Alemayehu, Getenet Beyene

**Affiliations:** 1 Department of Medical Laboratory Science, College of Health Science and Medicine, Dilla University, Dilla, Ethiopia; 2 School of Medical Laboratory Sciences, Institution of Health Sciences, Jimma University, Jimma, Ethiopia; 3 Armauer Hansen Research Institute, Addis Ababa, Ethiopia; 4 Institution of Biotechnology, Addis Ababa University, Addis Ababa, Ethiopia; Kohat University of Science and Technology, PAKISTAN

## Abstract

**Background:**

Shigellosis is an acute gastroenteritis infection and one of Ethiopia’s most common causes of morbidity and mortality, especially in children under five. Antimicrobial resistance (AMR) has spread quickly among *Shigella* species due to inappropriate antibiotic use, inadequacies of diagnostic facilities, and unhygienic conditions. This study aimed to characterize *Shigella sonnei (S*. *sonnei)* using whole genome sequence (WGS) analysis in Addis Ababa, Ethiopia.

**Methods:**

The raw reads were quality-filtered and trimmed, and a minimum length of 50bp was retained and taxonomically classified using MiniKraken version 1. The whole genome data were aligned with Antibiotic Resistance Gene (ARG) sequences of the Comprehensive Antibiotic Resistance Database (CARD) by Resistance Gene Identifier (RGI). Plasmids were analyzed using the PlasmidFinder tool version 2.1. Additionally, AMR and virulence genes were screened at the Centre for Genomic Epidemiology (CGE) web-based server.

**Results:**

All isolates in our investigation contained genes encoding *blaEC-8* and *blaZEG-1*. Here, 60.7% of the isolates were phenotypically sensitive to cefoxitin among the *blaEC-8* genes detected in the genotyping analysis, whereas all isolates were completely resistant to amoxicillin and erythromycin phenotypically. The study also identified genes that conferred resistance to trimethoprim (dfrA). Plasmid *Col156* and *Col (BS512)* types were found in all isolates, while *IncFII* and *Col (MG828)* plasmids were only identified in one isolate.

**Conclusion:**

This study found that many resistant genes were present, confirming the high variety in *S*. *sonnei* strains and hence a divergence in phylogenetic relationships. Thus, combining WGS methods for AMR prediction and strain identification into active surveillance may be beneficial for monitoring the spread of AMR in *S*. *sonnei* and detecting the potential emergence of novel variations.

## Background

*Shigella*, a Gram-negative bacterium from the Enterobacteriaceae family, is the second most prevalent cause of diarrheal mortality among individuals of all ages and a major cause of diarrheal sickness [1, 2]. More than 200,000 people die from shigellosis each year, with Low -and Middle-Income Countries (LMICs) bearing the majority of this burden [3]. *Shigella* is primarily transmitted by contaminated food and drink, as well as direct contact between humans. Typically, fever, tiredness, anorexia, and malaise indicate the beginning of a sickness. It can cause moderate diarrhea, severe dysentery with bloody stools, and even mortality from dehydration, particularly in vulnerable individuals such as small children, the elderly, and those with impaired immune systems [4]. Under five children are especially at risk from acute diarrhea, which is commonly defined as having at least three loose stools in 24 hours [5]. Sociocultural factors, poor quality drinking water, lack of formal education, and low hygiene levels are known risk factors for shigellosis, particularly in children under the age of five in developing nations [6, 7]. In comparison to other causes of gastroenteritis, *Shigella* is a highly contagious microorganism: It only takes 10 bacilli to cause an infection [8]. The most prevalent *Shigella* species in developed countries is *S*. *sonnei*. In contrast, *S*. *flexneri* is predominant in developing countries, while *S*. *dysenteriae* and *S*. *boydii* are rarely isolated [9]. *Shigella flexneri* (*S*. *flexneri*) is dominant in Africa and Asia, although *S*. *sonnei*, the most dominant species in South America, was the predominant isolate in one study in Ethiopia [10]. This variability may depend on different disease epidemiology between study sites. The prevalence of *Shigella* species reports varies in different studies due to factors that may have occurred during observation and measurement or that are linked to study methods and techniques [11]. Identification of the circulating bacteria is essential for treatment. The disease is complicated by a high rate of drug resistance to the commonly used antibiotic agents in different regions [9]. Antimicrobial resistance (AMR) pattern differs from place to place and between two regions in the same place [12]. The increasing prevalence of multi-drug resistance (MDR) to *Shigella* species is a serious threat, especially in Ethiopia with health and nutritional problems. There was identified MDR among several *Shigella* species isolated from acute diarrheal patients [13]. Irrespective of the serogroup/ serotype, most of the strains carried similar genes encoding resistance to specific antimicrobials. Most studies used standard culture to detect *Shigella* infection, with only a few studies using molecular methods, which can triple the detection rate by detecting lower-burden infections [14]. In Ethiopia, laboratory identification of *Shigella* species is nearly entirely dependent on culture and biochemical tests. However, one of the issues that diagnostic microbiology laboratories encounter is identifying *Shigella* strains from *E*. *coli*, which may be attributable to the fact that *E*. *coli* and all four *Shigella* species have very tight DNA-DNA connections [15]. Medication resistance based on genome-derived AMR data may be phenotypically susceptible to the linked antimicrobials [16]. *Shigella* species may now be identified using biochemical tests, and suspected colonies are confirmed by serotyping [15] with commercially available antisera. Serological identification of bacterial strains that create *Shigella*-like colonies on selective agar plates and biochemical tests show cross-reactivity with other bacteria such as *E*. *coli* and *Shigella*-specific antisera [17]. Polymerase chain reaction (PCR) test on the *IpaH* gene assay was utilized to identify *Shigella* species and Intro- invasive *E*. *coli* (EIEC) with excellent specificity [18]. However, the approach has no benefit in distinguishing *Shigella* from EIEC strains due to their identical virulence genes. Almost all molecular approaches fail to observe the evolutionary relationships between *Shigella* strains obtained within the country and those discovered outside. Because of this researchers are currently switching to whole genome sequencing (WGS) due to its higher resolution compared to previous methods (e.g., pulsed-field gel electrophoresis (PFGE) [19]. The sequencing data also give a higher level of strain differentiation and precision than any subtyping approach previously employed for epidemic identification and investigation. Thus, practically all characterization of *Shigella* species in the public health laboratory can be replaced by WGS employing commercial and web-based tools, including a serotype identification technique, AMR, plasmid, and virulence prediction tools for the study of *Shigella* and other microorganisms [20]. Despite the high incidence of shigellosis, there are limited data on the WGS analysis of Shigella species in Ethiopia, necessitating more inquiry. Therefore, this work was intended to investigate the genetic characteristics of S. sonnei utilizing WGS analysis in clinical samples in Addis Ababa, Ethiopia.

## Methods

### Study sites, sample collection, and bacterial identification

The study was conducted in stored isolates collected from four public health facilities from June 2021 to April 2022. *Shigella* growth was identified from other lactose-negative suspected colonies on Mac-Conkey agar (MAC) and xylose lysine deoxycholate (XLD) agar plates by unique colony morphology after subculturing and overnight incubation at 37°C. Additionally, biochemical tests were then utilized to identify the *Shigella* species. Antimicrobial susceptibility testing was also performed using a single-disk diffusion technique described in the previous phase-one study [21].

### Serogroups

*Shigella* serogroups were identified through the slide agglutination method. *Shigella* species do not produce flagellins or capsular antigens, hence their antigenic characterization is dependent on somatic antigens (O-antigens) using serological methods only. The slide agglutination test with polyvalent commercially available antisera was used to serogroup *Shigella* isolates.

### DNA extraction, library preparation, and whole genome sequencing(WGS)

The stored isolates were sub-cultured on nutrient agar (NA) media (Oxoid Ltd., Hampshire, UK) at 37°C via overnight incubation and the next day a pure colony was sub-cultured on nutrient broth (NB) (Oxoid Ltd., UK) at 37°C overnight. Then 200 mL was centrifuged at 5000 rpm, after which the sediment was ready for DNA extraction. DNA ex-traction was performed using the Qiagen DNA micro kit (QIAGEN, Hilden, Germany) according to the manufacturer’s instructions. DNA concentration was measured in a Qubit 4.0 fluorometer with the Qubit dsDNA HS Assay kit (Thermo-Fisher, Mumbai, MA, USA), and DNA purity was determined using the A260/A280 purity ratio. The target DNA con-centration was at or greater than 10 ng/μL, and the purity ratio was greater than 1.8 but less than 2. Each isolate’s DNA was processed for sequencing using the Nextera Flex DNA Library Preparation Kit (Illumina, San Diego, CA, USA) according to the manufacturer’s instructions. Isolates were sequenced using the Illumina NextSeq550 (Illumina, Singapore) technology at the Ethiopian Public Health Institute (EPHI) in conjunction with the Armauer Hansen Research Institute (AHRI). A high-output 300-cycle kit paired-end (PE) 149 bp long reads was produced.

### WGS data analysis of *S*. *sonnei* strains

The quality of the paired-end raw reads was assessed using the standard FAST-QC tools. Subsequently, trimming and filtering were performed to eliminate sequences from the beginning and end of reads and remove other adapter contamination and reads with low base call quality, utilizing trimmomatic tools. For further analysis, only high-quality paired-end reads with a Phred quality score greater than 20 and a minimum length of 50 base pairs were retained. Species confirmation was accomplished through gold standard Kmer-based taxonomy using MiniKraken version 1 (https://ccb.jhu.edu/software/kraken, (accessed on 22 May 2024)). Alignment was performed using the BWA-MEM algorithm, and mapping quality was evaluated using Samtools stats, assuring a depth of coverage larger than 20× and genome coverage greater than 85% for subsequent analysis. Variants were called using bcftools (http://samtools.github.io/bcftools/, (accessed on 22 May 2024)), with a subsequent filtration of SNPs to construct phylogenetic trees. The phylogenetic tree was constructed using maximum likelihood models with a bootstrap value of 1000 replications.

### Determination of AMR, virulence genes of isolates from whole genome data

Using genomic data, we employed an integrative method to identify plasmid, virulence, and antimicrobial resistance (AMR) genes. Firstly, the entire genome sequences were compared against Antibiotic Resistance Gene (ARG) sequences from the Comprehensive Antibiotic Resistance Database (CARD) using the Resistance Gene Identifier (RGI, v5.1.1). Genes exhibiting 90–100% identity and coverage were classified as perfect or strict matches, with any ambiguities excluded. Reference sequences for acquired resistance genes were sourced from CARD, and refined using the AMRFinderplus database. Additionally, genes screened using the ResFinder 2.0 database (https://cge.food.dtu.dk/services/ResFinder, (accessed on 2 June 2024)). PlasmidFinder detected plasmids; VirulenceFinder 1.5 screened virulence gene by CGE web-based server (https://cge.food.dtu.dk/services/VirulenceFinder/, (accessed on 4 June 2024)) with a threshold of 90% identity and 60% minimum length. This methodology ensured comprehensive assessment, aligning reads to a reference database of acquired genes [22, 23].

### Quality control

The standard operating procedure (SOP) and Clinical Laboratory Standards Institute (CLSI) were used throughout the procedures. In addition, the sterility of the culture media was checked frequently by incubating the prepared culture media at 37°C overnight and checking for growth. American Type Culture Collection (ATCC) reference strains were used to test the performance. The temperature utilized to store the disks, materials, and regents followed the manufacturer’s instructions. The isolates were held at -80°C, whereas the DNA was kept at -20°C. All DNA and library preparation methods followed standard and manufacturer’s instructions.

### Ethics statement

The investigation was conducted using previously archived study samples and received ethical approval from the Jimma University Institute of Health Institutional Review Board (IRB) (IHRP6/1092/2021). AHRI and EPHI also approved the study. As stated in our previous study [21], the mothers/caregivers of the children provided informed, voluntary, written, and signed consent after the study’s purpose was described to them in detail. Parents/guardians of the children were also informed about the confidentiality of the information acquired and their complete right to refuse or withdraw from participation in the research.

## Results

### *Shigella* isolates

**Fig 1:**
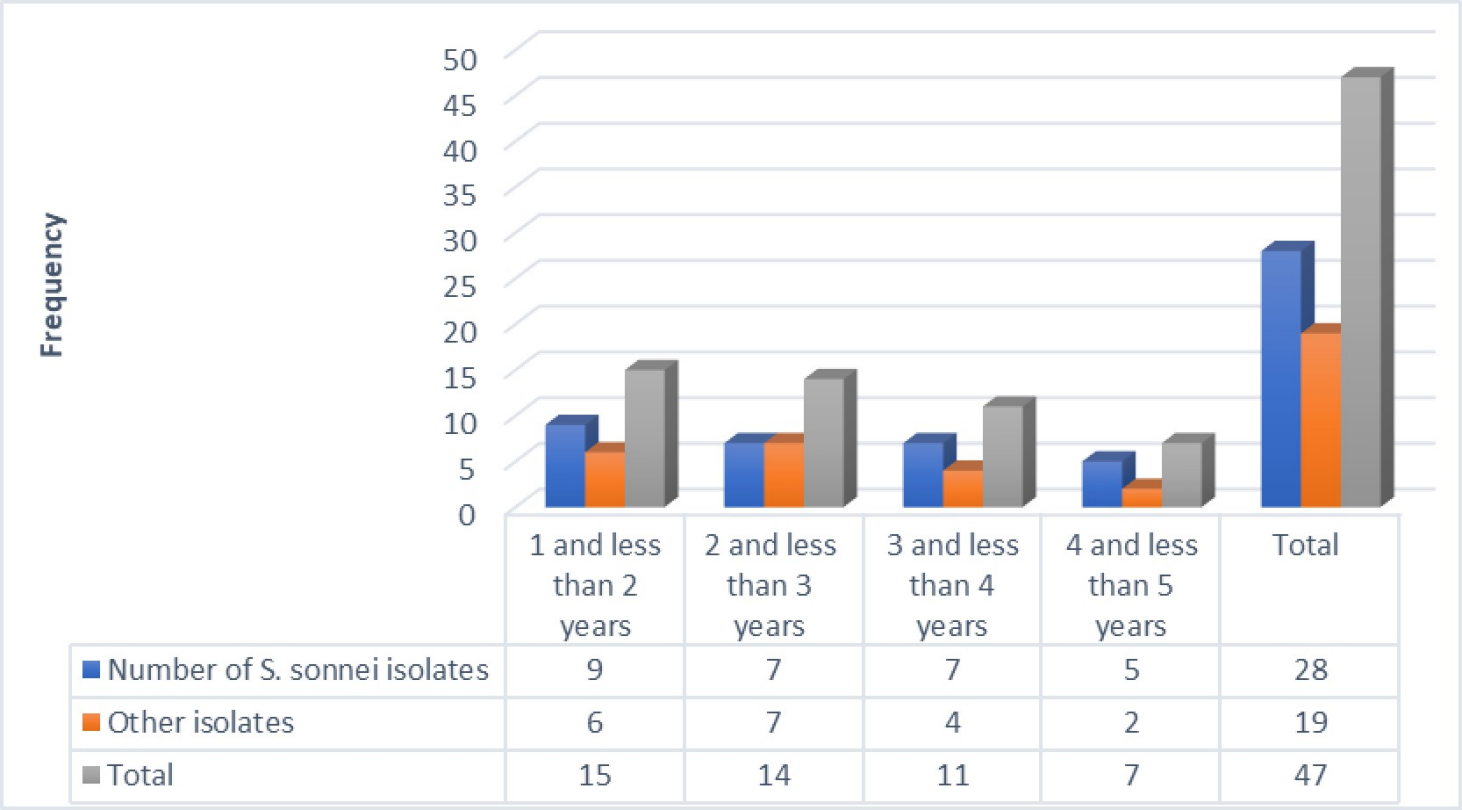
Distribution of isolates by age category in clinical isolate samples from four public health facilities in Addis Ababa, Ethiopia.

From 534 stool specimens, 47 (8.8%) *Shigella* species were identified using culture and biochemical assays, as stated in the earlier phase-one investigation (S1 Table). The age group of one to less than three years old had the highest percentage of infections (5.4%); however, no pathogen was isolated from children under one year. Of the 47 *Shigella* species, 31 were serologically identified as *S*. *sonnei* and 16 as *S*. *flexneri*. Of the isolates, 28 were *S*. *sonnei* (Fig 1), which were selected for further study utilizing WGS data using the reference strain accession NZ_CP055292.1. All isolates were *S*. *sonneiSE6*.*1* strains and had Query_Coverage with 87.61 to 92.43.

### Comparison between phenotypic and genotypic AMR profile of *Shigella sonnei*

Phenotypically, in terms of susceptibility, among the 47 Shigella species, 100%, 93.6%, 80.9%, 72.3%, and 57.5% of the isolates were sensitive to norfloxacin, nalidixic acid, ciprofloxacin, gentamicin, and cefoxitin, respectively. Ampicillin resistance reached 93.6%. However, all isolates were completely resistant to amoxicillin and erythromycin (S1 Table). Here, 7 and 17 isolates showed discordant results for cotrimoxazole (trimethoprim) and cefoxitin, respectively, between the phenotype and genotype (Table 1). The majority were predicted to be susceptible based on genome-derived AMR data. Still, these were phenotypically resistant to the corresponding antimicrobials (S1 Table), so all were classified as major errors (genotypically susceptible but phenotypically resistant).

**Table 1:** Evaluation of genotypic analysis for predicting phenotypically susceptible patterns of all viable isolates (n = 28).

Isolates (%)	Antimicrobial Tested	Drug Class	Resistance Status		Resistant Gene	Accession
			Phenotype	Genotype		
**B26, B92, B129, B182, B356, B408, B442 (25)**	Cotrimoxazole (trimethoprim)	Folate synthesis inhibitors	Susceptible	Resistant	*dfrA*	KU546748.1
**B25, B26, B52, B71, B82,B92, B108,B115, B143,B178, B182,B245, B311, B322; B382, B408, B442 (60.7)**	Cephalosporin (cefoxitin)	Beta-lactam	Susceptible	Resistant	*blaEC-8*	NG_049086
**All**	Unknown beta-lactam	Beta-lactam	Unknown	Resistant	*blaZEG-* *1* ^ₐ^	AY265891

^ₐ^ The identified *blaZEG-1* gene had been associated with unknown beta-lactam antibiotics listed in the CGE web-based server. Every isolate had identified the *blaZEG-1* genotype gene. This gene was not detected in the tested beta-lactam drugs, such as cefoxitin, ampicillin, and amoxicillin.

### Resistance to beta-lactams and folate synthesis inhibitors

All of the isolates in our study had genes that encoded beta-lactam drugs, particularly *blaEC-8* and *blaZEG-1*. Here, 60.7% of the isolates were phenotypically sensitive to cefoxitin among the *blaEC-8* genes detected in the genotyping analysis. All isolates possessed genes that made them resistant to trimethoprim. Of the genotype resistance isolates, 25% were phenotypically susceptible to trimethoprim. The gene isolates that conferred resistance to trimethoprim had the dfrA gene (Table 1).

### Antibiotic resistance genes (ARGs) in *S*. *sonnei*

We found 52 types of ARGs in the whole genome data for each *S*. *sonnei* isolate. Besides a total of 224 perfect ARG matches, a further 1232 hits were classified as strict and met the criteria of having 100% and 90–99.9%, respectively for coverage and sequential identity. Resistance gene identifier(RGI) criteria were set to predict perfect, strict, and complete genes only, which returned 8 perfect hits and 44 strict hits with no loose hits in each isolate. The resistance mechanism for the perfect RGI criteria includes seven genes *cpxA*, *TolC*, *mdtE*, *emrR*, *emrY*, *marA*, and *H-NS* involved in antibiotic efflux, and one ARG gene, i.e., *PmrF* involved in antibiotic target alteration, while *marA* also involved in reduce permeability to antibiotics in the isolates. The resistance mechanism in strict hit includes 35 genes involved in antibiotic efflux, one gene that has reduced permeability to an antibiotic, one involved in antibiotic inactivation, and thirteen genes involved in antibiotic target alteration (Table 2). All isolates had *E*. *coli AcrAB-TolC* with *AcrR* mutation conferring resistance to ciprofloxacin, tetracycline, and ceftazidime while *E*. *coli AcrAB-TolC* with *MarR* mutations conferring resistance to ciprofloxacin and tetracycline. Additionally, *E*. *coli GlpT* with mutation conferring resistance to fosfomycin and *E*. *coli EF-Tu* mutants conferring resistance to Pulvomycin were detected. The proportion of resistance mechanisms was calculated based on the ARG diversity. The dominant resistance mechanism of identified ARGs was antibiotic efflux (71.2%), antibiotic target alteration (23.7%), reduced permeability to antibiotics (3.4%), and antibiotic inactivation (1.7%).

**Table 2:** Identified Antibiotic Resistance Genes (ARGs) and their resistance mechanisms in perfect and strict hits of S. sonnei in Addis Ababa, Ethiopia (n = 28).

AMR gene family	Strict hits(90–99.9%)		Perfect hits (100%)		Resistance Mechanism
	n	Type of genes	n	Type of genes	
**General Bacterial Porin with reduced permeability to beta-lactams**	1	*E*. *coli* soxS with mutation conferring antibiotic resistance	1	*MarA*	reduced permeability
**antibiotic-resistant GlpT**	1	*E*. *coli* GlpT with mutation conferring resistance to fosfomycin			antibiotic target alteration
**elfamycin resistant EF-Tu**	2	*E*. *coli EF-Tu* mutants conferring resistance to Pulvomycin(2)			antibiotic target alteration
**Penicillin-binding protein mutations conferring resistance to beta-lactam antibiotics**	1	*H*. *influenzae* PBP3 conferring resistance to beta-lactam antibiotics			antibiotic target alteration
**small multidrug resistance (SMR) antibiotic efflux pump**	3	*E*. *coli emrE*, *K*. *pneumoniae KpnE*(2)			Antibiotic efflux
**undecaprenyl pyrophosphate-related proteins**	1	*BacA*			antibiotic target alteration
**pmr phosphoethanolamine transferase**	3	*eptA*, *ArnT*, *ugd*	1	*PmrF*	antibiotic target alteration
**ampC-type beta-lactamase**	1	*E*. *coli* ampC beta-lactamase			antibiotic inactivation
**Van ligase**	1	*VanG*			antibiotic target alteration
**glycopeptide resistance gene cluster**	1	*VanG*			antibiotic target alteration
**resistance-nodulation-cell division (RND) antibiotic efflux pump**	19	*acrB*, *gadX*,*mdtF*, *mdtC*,*mdtB*, *mdtA*, *baeR*, *CRP*, *AcrE*, *AsrS*. *rsmA*, *acrD*, *evgS*, *evgA*, *E*. *coli acrA*, *E*. *coli acrR* with mutation conferring antibiotic resistance, *E*. *coli soxR* with mutation conferring antibiotic resistance, *E*. *coli soxS* with mutation conferring antibiotic resistance, *E*. *coli marR* mutant conferring antibiotic resistance	5	*cpxA*,*mdtE*, *TolC*,*marA*, *H-NS*	Antibiotic efflux
**KdpDE**	1	*KdpE*			
**ATP-binding cassette (ABC) antibiotic efflux pump**		*msbA*, *YojI*, *E*. *coli soxR* with mutation conferring antibiotic resistance, *E*. *coli soxS* with mutation conferring antibiotic resistance	1	*TolC*	Antibiotic efflux
**major facilitator superfamily (MFS) antibiotic efflux pump**	14	*mdtH*, *mdtG*, *E*. *coli mdfA*, *leuO*, *mdtN*, *mdtM*, *mdtP*, *emrA*, *emrB*, *evgS*, *evgA*, *emrK*, *E*. *coli soxR* with mutation conferring antibiotic resistance, *E*. *coli soxS* with mutation conferring antibiotic resistance	4	*TolC*, *emrR*, *emrY*,*H-NS*	Antibiotic efflux

### Drug class and their ARGs

The finding that ARGs may compromise the efficacy of various antibiotic classes (Table 3) raises some clinical concerns. The identified ARGs may have affected many of the most highly prioritized antibiotics. For human *Shigella* infection treatment in Ethiopia, diaminopyrimidine(trimethoprim or cotrimoxazole), cephalosporins (cefixime, ceftriaxone), macrolides (azithromycin), penicillins of various categories (ampicillin, amoxicillin), quinolones (nalidixic acid, ciprofloxacin, norfloxacin), aminoglycosides (streptomycin, gentamicin) could be affected by the ARGs identified in the isolates. Antibiotic resistance genes (ARGs) were also discovered, confirming resistance to disinfectants and antiseptics for *S*. *sonnei* in perfect hits such as *TolC* and *marA* genes. In addition, multiple resistance genes to disinfectants and antiseptics were found in the strict hits, including *E*. *coli acrR* with mutation conferring multidrug antibiotic resistance gene.

**Table 3:** Drug class and ARGs in perfect and strict hits of *S*. *sonnei* in Addis Ababa, Ethiopia (n = 28).

Drug class	Strict hits	Perfect hits
	Resistant genes	Resistant genes
**Penem**	*E*. *coli soxS* with mutation conferring antibiotic resistance	*TolC; marA*
**Carbapenem**	*E*. *coli soxS* with mutation conferring antibiotic resistance	*TolC*, *marA*
**Monobactam**	*E*. *coli soxS* with mutation conferring antibiotic resistance	*MarA*
**Elfamycin**	*E*. *coli EF-Tu* mutants conferring resistance to Pulvomycin(2)	
**Diaminopyrimidine**	*RsmA*	
**Aminocoumarin**	*mdtC*. *mdtB*, *mdtA*,*baeR*	*TolC*, *cpxA*
**Cephamycin**	acrE, acrS, *E*. *coli soxS* with mutation conferring antibiotic resistance, *H*. *influenzae* PBP3 conferring resistance to beta-lactam antibiotics	*TolC; H-NS; marA*,
**Macrolide**	*K*. *pneumoniae KpnE(2)*, *E*. *coli emrE*, *gadX*, *mdtF*, *CRP*, *evgS*, *evgA*	*TolC*, *mdtE*,*H-NS*
**Peptide**	*K*.*pneumoniae KpnE(2)*, *eptA*, *bacA*, *ArnT*, *YojI*, *udg*	*TolC*, *PmrF*
**Lincosamide**	*MdtM*	
**Nucleoside**	*leuO*, *mdtM*, *mdtN*, *mdtP*	
**Glycopeptide**	*VanG*	
**Phenicol**	*E*. *coli acrA*, *acrB*, *acrS*, *mdtM*, *rsmA*, *E*. *coli acrR* with mutation conferring multidrug antibiotic resistance, *E*. *coli soxR* with mutation conferring antibiotic resistance, *E*. *coli soxS* with mutation conferring antibiotic resistance, *E*. *coli marR* mutant conferring antibiotic resistance	*TolC; marA*
**Rifamycin**	*E*. *coli acrA*, *acrB*, *acrS*, *K*. *pneumoniae KpnE(2)*, *E*. *coli acrR* with mutation conferring multidrug antibiotic resistance, *E*. *coli soxR* with mutation conferring antibiotic resistance, *E*. *coli soxS* with mutation conferring antibiotic resistance, *E*. *coli marR* mutant conferring antibiotic resistance	*TolC; marA*
**Penam**	*E*. *coli soxS* with mutation conferring antibiotic resistance, *E*. *coli* ampC beta-lactamase *acrB*, *gadX*, *mdtF*, *CRP*, *acrE*, *asrS*, *evgS*, *evgA*, *H*. *influenzae* PBP3 conferring resistance to beta-lactam antibiotics, *E*. *coli marR* mutant conferring antibiotic resistance, *E*. *coli soxR* with mutation conferring antibiotic resistance, *E*. *coli acrR* with mutation conferring multidrug antibiotic resistance, *E*. *coli acrR*,	*TolC; H-NS; mdtE; marA*
**Glycylcycline**	*E*. *coli soxS* with mutation conferring antibiotic resistance, *acrB*, *asrS*, *E*. *coli marR* mutant conferring antibiotic resistance, *E*. *coli soxR* with mutation conferring antibiotic resistance, *E*. *coli acrR* with mutation conferring multidrug antibiotic resistance, *E*. *coli acrR*	*TolC; marA*
**Cephalosporin**	*E*. *coli acrA*, *acrB*, *E*. *coli* ampC beta-lactamase, *AcrE*, *AcrS*, *K*. *pneumoniae KpnE(2)*, *H*. *influenzae* PBP3 conferring resistance to beta-lactam antibiotics, *E*. *coli acrR* with mutation conferring multidrug antibiotic resistance, *E*. *coli soxR* with mutation conferring antibiotic resistance, *E*. *coli soxS* with mutation conferring antibiotic resistance, *E*. *coli marR* mutant conferring antibiotic resistance	*TolC; H-NS; marA*
Aminoglycoside	*kdpE*, *acrD*, *baeR*, *Klebsiella pneumoniae KpnE*(2)	*TolC*, *cpxA*
**Disinfecting agents and antiseptics**	*acrB*, *leuO*, *mdtM*, *mdtN*,*mdtP*, *AsrS*, *E*. *coli mdfA*, *E*. *coli soxS* with mutation conferring antibiotic resistance, *E*. *coli marR* mutant conferring antibiotic resistance, *E*. *coli soxR* with mutation conferring antibiotic resistance, *E*. *coli acrR* with mutation conferring multidrug antibiotic resistance, *E coli acrR*, *K*. *pneumoniae KpnE*(2)	*TolC; marA*
**Tetracycline**	*acrB*, *AsrS*, *emrK*, *evgA*, *evgS*, *E*. *coli mdfA*, *E*. *coli soxS* with mutation conferring antibiotic resistance, *E*. *coli marR* mutant conferring antibiotic resistance, *E*. *coli soxR* with mutation conferring antibiotic resistance, *E*. *coli acrR* with mutation conferring multidrug antibiotic resistance, *E*. *coli acrR*, *Klebsiella pneumoniae KpnE*(2)	*TolC; emrY;* *H-NS; marA*
**Nitroimidazole**	*MsbA*	
**Phosphonic acid**	*mdtG*, *E*. *coli GlpT* with mutation conferring resistance to fosfomycin	
**Fluoroquinolone**	*mdtH*, *acrB*, *gadX*, *mdtM*, *mdtF*, *CRP*, *acrE*, *asrS*, *rsmA*, *emrB*, *emrA*, *evgA*, *evgS*, *E*. *coli soxS* with mutation conferring antibiotic resistance, *E*. *coli marR* mutant conferring antibiotic resistance, *E*. *coli soxR* with mutation conferring antibiotic resistance, *E*. *coli acrR* with mutation conferring multidrug antibiotic resistance, *E*. *coli acrR*	*TolC; mdtE; emrR;* *H-NS; marA*

### Plasmid analysis

Through the PlasmidFinder tool, we identified the presence of position in ref, with their percent identity related to plasmids in *S*. *sonnei* strains, as shown in Table 4. Plasmid *Col156* and *Col* (*BS512*) types were found in all isolates. The *IncB/O/K/Z* plasmid was discovered in all isolates except B27, B52, B92, and B311; however, the *IncFII* plasmid incompatibility element and *Col* (*MG828*) plasmids were only isolated in isolate B394.

**Table 4:** Plasmid distribution in *S*. *sonnei* isolates (n = 28).

Isolates ID (%)	Plasmid	Identity (%)	Position in Ref.	Coverage (%)	Accession
B25,B55,B71,B112, B115,B129,B143,B178,B184,B196,B416,B494(42.9)	*IncB/O/K/Z*	95.97	1…50	100	FN868832
	*Col156*	94.81	1…155	100	NC009781
	*Col*(*BS512*)	100	1…234	100	NC010656
B26(3.6)	*IncB/O/K/Z*	93.29	6…150	96.64	FN868832
	*Col156*	94.81	1…155	100	NC009781
	*Col*(*BS512*)	100	1…234	100	NC010656
B27,B52,B92,B311(14.3)	*Col156*	94.81	1. . .155	100	NC009781
	*Col*(*BS512*)	100	1. . .234	100	NC010656
B82(3.6)	*IncB/O/K/Z*	93.29	5. . .150	97.32	FN868832
	*Col156*	94.81	1. . .155	100	NC009781
	*Col*(*BS512*)	100	1. . .234	100	NC010656
B108,B182,B245,B382,B408,B442,B510(25)	*IncB/O/K/Z*	92.62	8. . .150	95.3	FN868832
	*Col156*	94.81	1. . .155	100	NC009781
	*Col*(*BS512*)	100	1. . .234	100	NC010656
B322(3.6)	*IncB/O/K/Z*	94.63	4. . .150	97.99	FN868832
	*Col156*	94.81	1. . .155	100	NC009781
	*Col*(*BS512*)	100	1. . .234	100	NC010656
B356(3.6)	*IncB/O/K/Z*	93.29	6. . .150	96.64	FN868832
	*Col156*	94.81	1. . .155	100	NC009781
	*Col*(*BS512*)	100	1. . .234	100	NC010656
B394(3.6)	*IncB/O/K/Z*	100	1.150	100	CU928147
	*Col156*	94.81	1. . .155	100	NC009781
	*Col*(*BS512*)	100	1. . .234	100	NC010656
	*IncFII_1_pSFO*	90.31	1. . .259	94.96	AF401292
	*Col*(*MG828*)	91.6	4. . .263	98.85	NC008486

### Virulence gene analysis

The existence of virulence genes was investigated using the *E*. *coli* database. The detection of virulence factors indicated little variations between strains and no genes producing *Shigatoxin*. The majority of the isolates included virulence genes such as *gad*, which were frequently detected, and which are important to the glutamate decarboxylase in the epithelial cells. Isolate B322 had one gad gene, B25, B55, B71, B92, B112, B184, B245, B356, B382, B416, and B510 had three gad genes, and the remaining had two gad genes. Other virulence genes found in the isolates include *csgA*, *fdeC*, *gad*(*3*), *fimH*, *hlyE*(*2*), *nlpl*, *lpfA*, *terC*(*2*), *yehA*, *yehB*, *yehC* and *yehD*, as are shown in Table 5.

**Table 5:** Virulence genes for *S*. *sonnei* isolates in Addis Ababa, Ethiopia(n = 28).

Virulence Gene	Position in Contag.	Protein Function	Accession
*csgA*	152198… 152653	Curling major subunit CsgA	CP069646
*fdeC*	929546… 933799	Intimin-like adhesin FdeC	AP010953
*gad*	4354291…4355691 and 2232338… 2233738	Glutamate decarboxylase	AP010953 and CU928160
*fimH*	1357284… 1357772	Type 1 fimbriae	NA
*hlyE*(*2*)	1074… 1977 and 4714745… 4715661	Avian E. coli haemolysin	ECU57430
*Nlpl*	2566573… 2567457	lipoprotein NlpI precursor	CP000243
*lpfA*	1975240… 1975812	Long polar fimbriae	AY646923
*terC*(*2*)	2945444… 2946157 and 2644155… 2645113	Tellurium ion resistance	CP007491 and MG591698
*yehA*	3703267… 3704301	Outer membrane lipoprotein, YHD fimbriael cluster	CP042934
*yehB*	3700773… 3703253	Usher, YHD fimbriael cluster	CP042934
*yehC*	3700083… 3700757	Chaperone, YHD fimbriael cluster	CP042934
*yehD*	3699460… 3700002	Major pilin subunit, YHD fimbriael cluster	CP042934

### Phylogenetic analysis

**Fig 2:**
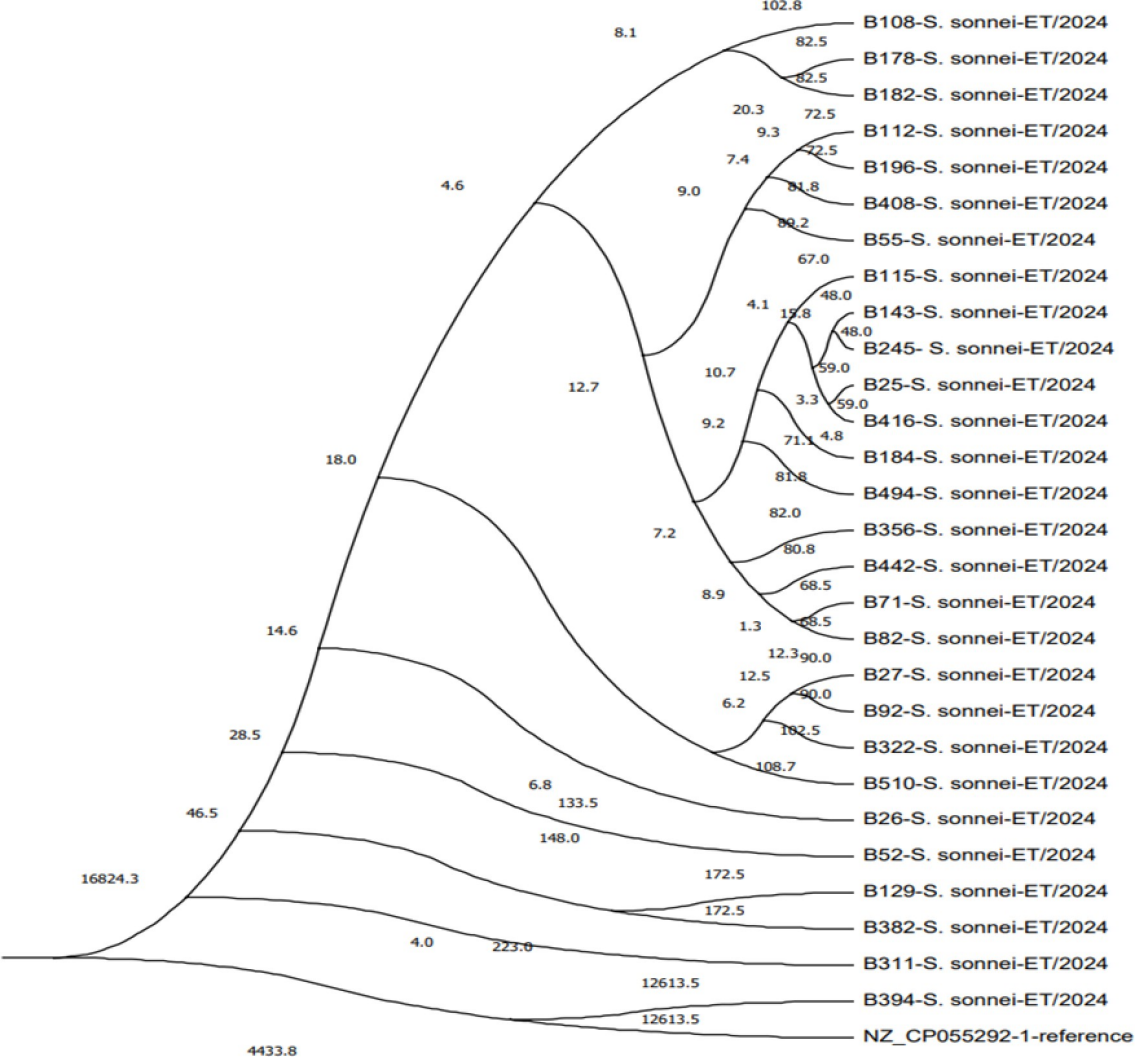
Phylogenetic topology tree. Phylogenetic tree based on SNP analysis in CSI Phylogeny 1.4 analyzing 28 *S*. *sonnei* genomes from strains obtained at 4 public health facilities in Ethiopia. The tree file with the Newick extension produced by CSI Phylogeny 1.4 was utilized.

**Fig 3:**
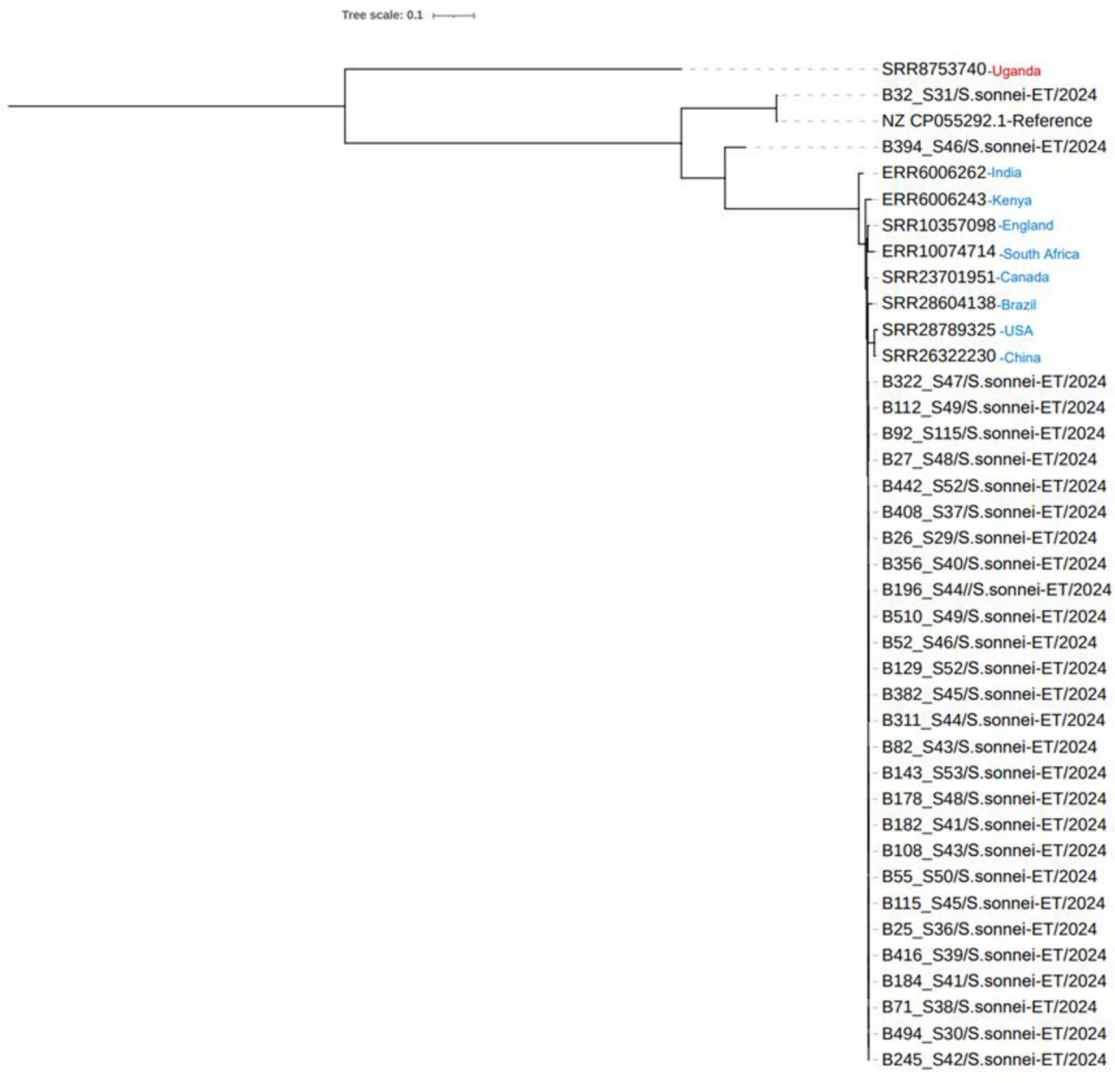
Phylogenetic relationship with the global isolates. The generated tree shows the relatedness of local and global isolates. To perform SNP calling, sequencing data for the nine global *S*. *sonnei* strains (red and blue colors) were retrieved from the NCBI SRA and entered into SNVPhyl alongside the local isolates.

We sequenced the genomes of the 28 isolates and conducted a single-nucleotide polymorphism (SNP) phylogenetic analysis using the data (Figs 2 and 3). We chose and downloaded the genome sequences of 28 isolates to assess the similarity between strains. We discovered that they were divided into seven major phylogenetic groupings (PGs) and more diverse *S*. *sonnei* strains (PG1-PG7). The phylogenetic tree revealed that most strains were clustered in one group with PG6 strains, which included 22 strains, indicating a high level of genomic similarity between them. Six isolates (B26, B52, B382, B129, B311, and B394) deviated from the PG6 group, indicating distinct origins. The phylogenetic tree obtained by mapping our 28 *S*. *sonnei* paired-end reads against a reference genome showed the genetic diversity of the strains, with strains B108, B178, and B182 being the most phylogenetically distant. However, strain B394 appeared to be closely related to the reference. Overall, the phylogenetic tree became more diversified and revealed that strains were divided into several distinct groups, implying numerous origins.

### Phylogenetic relationships of *S*. *sonnei* globally

Single-nucleotide polymorphism (SNP) calling was performed, and a maximum-likelihood tree was constructed to illustrate the relatedness of these *S*. *sonnei*; grouping by strain geographic origin was interpreted as the key driver of phylogenetic segmentation. *S*. *sonnei* isolated from Uganda varied considerably from the other isolates (Fig 3). Additional branching occurred in one isolate (B394). Most of the remaining 35 isolates from closely related strains formed separate, smaller clusters. However, overall grouping appears to be irrespective of geographic origin, since one large cluster includes both international and local isolates.

## Discussion

Shigellosis is the leading cause of death and diarrhea in children under the age of five in underdeveloped countries [24]. Studies on the molecular characterization of *Shigella* isolates are extremely rare, particularly in underdeveloped countries with limited resources, such as Ethiopia. Although *Shigella* and *E*. *coli* have similar features, there is sometimes a clinical or public health requirement to distinguish between these infections since they have distinct entities in epidemiology and clinical practice. New *Shigella* strains that do not agglutinate with commercially available antisera are becoming more common [25]. *Shigella* genome sequencing was previously examined and shown to be divided into seven phylogenetic groupings (PGs) [26, 27]. We selected and downloaded the full genome sequences of 28 isolates to study the similarity between strains, and we observed that they were split into seven major PG and more diversified *S*. *sonneiSE6-1* strains (PG1-PG7). The phylogenetic tree could determine the connection between isolates by constructing clades and branches [22]. In the current study, the phylogenetic tree reveals that most of the *S*. *sonneiSE6-1* strains were associated with PG6 strains, which contained 22 strains and shared a high level of genomic similarity. On the other hand, six isolates branched away from the PG6 group, indicating distinct origins. Generally, the phylogenetic tree was more diversified, demonstrating that strains were classified into numerous unique groups, representing several origins.

The global relatedness of *S*. *sonnei* identified from Uganda differs significantly from other isolates. In the current study, we observed additional branching in one of the isolates. There was no indication of geographical clustering because the groups contained local and global isolates acquired [23]. Almost all of the remaining 35 isolates from closely related strains formed separate, smaller clusters. However, overall grouping appears to be irrespective of geographic origin, as one large cluster includes both international and local isolates.

Plasmids containing AMR determinants in clinical strains are a serious global concern [28]. The plasmid *Col156 and Col(BS512)* detection are the genetic components that recognize plasmids containing the colicin E production, immunity, and lysis system [29]. The current investigation revealed that *Col156* and *Col(BS512)* dominated the detected isolates, with 94.81% and 100% identity, respectively. The presence of incompatible plasmids, specifically the *IncF* plasmid, has been linked to the global emergence of clinically relevant ESBLs and multiple AMR determinants [30]. The *IncFII* plasmid was only identified in the B394 isolate however *IncB/O/K/Z* plasmid was detected in most isolates in this finding. Levere and his colleagues discovered that azithromycin resistance was eventually acquired, mostly via *IncFII* plasmids. Various ESBL genes are carried by different plasmids (IncFII, IncB/O/K/Z) or even incorporated into the chromosome, and they encode resistance to third-generation cephalosporins [31].

The primary goal of this study was to identify genes associated with antibiotic resistance. Consequently, the resistance pattern phenotypic and the genes involved in AMR were examined and contrasted. In contrast to the genetic data, most isolates showed greater phenotypic resistance to antimicrobials tested, as described in the previous study [21]. Since resistance genes are frequently plasmid-mediated, differences may result from some plasmid loss during storage [16]. When phenotypic resistance occurs without gene detection, it suggests that resistance may arise from other mechanisms. On the other hand, genotypes present resistance genes without phenotypic expression, indicating that AMR genes are not expressed [30].

The pathogenesis of *Shigella* is associated with several virulence factors found in chromosomes or large virulent plasmids that carry genes essential for survival within cells and host cell invasion. Only a few researchers, meanwhile, have tried to describe their molecular virulence profiles. Based on a recent study [30], Shigella virulence genes are linked to several clinical symptoms, including severe stomach discomfort and bloody diarrhea. The authors also emphasized that resistance to more antimicrobials was linked to virulence genes in greater quantities. While analyzing the various genes, it was discovered that the separated virulence genes are involved in pathogenicity. A study [32] found a significant prevalence of pathogenicity-associated islands with the *fimH* gene, providing evidence for their involvement. Similarly, another study found the existence of specific virulence genes, such *as iha*, *lpfA*, *yeh*(*A-D*), and *gad*, controlling adhesion to host cells [33].

This study also found several genes that lead to a high degree of drug class resistance pattern. Among the resistance genes discovered, *mdtM* and *E*. *coli acrR* with the mutation were linked to antibiotic efflux. This finding further demonstrated that the same genes were responsible for multidrug resistance and enabled the organism to withstand stress conditions involving an alkaline environment, higher antibiotic concentrations, and external pH [33]. Furthermore, *TolC*, *mdtE*, and *H-NS* genes in perfect hits, and *emrE*, *gadX*, *mdtF*, *CRP*,*evgS*, and *evgA* genes in strict hits which were observed to be involved in antibiotic efflux, were found to be responsible for causing resistance to macrolide antibiotics [34]. The gene complements of *E*. *coli* and *Shigella* species are very similar, and these species cannot be separated into two groups, since they have a common gene pool in gene phylogenetic trees [35]. The results presented demonstrate that the *S*. *sonnei* carried significant amounts of ARGs, and, those genes may also be mobile, having possible consequences on the antibiotic treatment efficacy. As seen above, and as described in other publications [34], there is still a great deal of variation in details that need to be clarified by the interpretation of ARGs. Nonetheless, the possibility that the identified ARGs may impair the efficacy of several antibiotic classes raises clinical concerns. According to the most recent Center for Disease Control (CDC) data on antimicrobial usage in the United States, the most often provided substances are amoxicillin (penam), azithromycin (macrolide), aminoglycoside, amoxicillin, and clavulanic acid (penam, enhanced activity), cephalexin (cephalosporin), and doxycycline (tetracycline) [36]. Furthermore, according to the most recent WHO report on the use of antibiotics worldwide, the most often prescribed oral medications are amoxicillin (penam), ciprofloxacin (fluoroquinolone), sulphamethoxazole, and trimethoprim; in four African countries surveyed, the most often used parenteral medications are ceftriaxone (cephalosporin), gentamicin (aminoglycoside), and benzylpenicillin (penam) [37]. The detection of ARGs may have an impact on several of the most high-priority antibiotics. In the current study, based on the RGI criteria *S*. *sonnei* was confirmed resistant to almost all available antibiotics for *Shigella* species. Shigellosis was initially treated with sulfonamides and tetracycline, then with ampicillin, trimethoprim/sulfamethoxazole, and nalidixic acid [28]. These medications are no longer indicated unless susceptibility is established owing to the advent of resistant strains. The latest WHO report on critically important antimicrobials (CIA) for human *Shigella* infection treatment includes folate synthesis inhibitors (trimethoprim or cotrimoxazole), cephalosporins (ceftriaxone, cefixime), macrolides (azithromycin), penicillins (ampicillin, amoxicillin), quinolones (nalidixic acid, ciprofloxacin, norfloxacin), and aminoglycosides (streptomycin, gentamicin) [37]. Additionally, disinfectants and antiseptics were used to minimize the spread of the *Shigella* infection. In line with the study in Egypt [38], the current findings suggested the emergence of *S*. *sonnei* exhibited multi-resistance to either antibiotics (especially ESBL-producing strains) or disinfectants.

It is significant to note that all isolates had the gene *H-NS*, which is critical in the global gene regulation of many bacteria, including this species. The *H-NS* inhibits the expression of several genes, and its loss enhances AMR while decreasing drug accumulation. Even though this gene is contained in CARD, its functional impact is opposite to that of ARGs [39]. The ARGs not only diminish the efficacy of antibiotic therapy on *S*. *sonnei* but are also passed to other pathogenic bacteria in the consumer’s body [40], potentially reducing the efficiency of antibiotic therapy on disorders that include other pathogenic bacteria.

## Conclusion

In conclusion, the current investigation improves our understanding of *S*. *sonnei* linked with diseases reported to Ethiopian public health authorities and emphasizes the importance of WGS in explaining the phenotypic–genotypic AMR association. All isolates in our investigation contained genes encoding *blaEC-8* and *blaZEG-1*. Here, 60.7% of the isolates were phenotypically sensitive to cefoxitin among the *blaEC-8* genes detected in the genotyping analysis, whereas all isolates were completely phenotypically resistant to amoxicillin and erythromycin. The study also identified gene carriers conferring resistance to trimethoprim (*dfrA*). Plasmid *Col156 and Col* (*BS512*) types were found in all isolates. Additionally, the identified ARGs were discovered to be involved in antibiotic target changes, and their overexpression led to decreased permeability and antibiotic efflux. This WGS study found that many genes were present, confirming more variety in *S*. *sonnei* strains and hence greater divergence in phylogenetic relationships. Limiting the findings to *S*. *sonnei* made characterizing other *Shigella* species that cause diarrhea more difficult. The study only covered *S*. *sonnei*. However, combining WGS approaches for AMR prediction and strain identification into active surveillance may be useful for tracking the spread of AMR in *S*. *sonnei* and detecting the possible development of new variants. Furthermore, we recommend that researchers examine the origins of AMR disparities and implement strategies to minimize ARG spread in Ethiopia.

## Supporting information

S1 Table Antimicrobial resistance pattern of Shigella isolated from stool cultures among under five diarrheic children in selected health centers, Addis Ababa (June 2021 to April 2022). S, Sensitive; I, Intermediate; R, resistant. Notice: any results related to phase one study can be accessible at: Ayele, B.; Mekonnen, Z.; Sisay Tessema, T.; Adamu, E.; Tsige, E.; Beyene, G. Antimicrobial Susceptibility Patterns of Shigella Species among Children under Five Years of Age with Diarrhea in Selected Health Centers, Addis Ababa, Ethiopia. Can. J. Infect. Dis. Med. Microbiol. 2023, 2023(1), 5379881.
(DOCX)

S2 Table Performance standards for antimicrobial susceptibility testing of the commonly prescribed antibiotics (adopted from CLSI and EPHI national clinical bacteriology and mycology reference laboratory) (CLSI, 2021). (DOCX)

S1 File FASTA data file. (RAR)

## References

[1] Hausdorff WP, Anderson IV JD, Bagamian KH, Bourgeois AL, Mills M, Sawe F, et al. Vaccine value profile for Shigella. Vaccine. 2023; 41:S76–S94. doi: 10.1016/j.vaccine.2022.12.037 37827969

[2] Prudden H, Hasso-Agopsowicz M, Black R, Troeger C, Reiner R, Breiman R, et al. Meeting Report: WHO Workshop on modelling global mortality and aetiology estimates of enteric pathogens in children under five. Cape Town, 28–29th November 2018. Vaccine. 2020; 38(31):4792–4800. doi: 10.1016/j.vaccine.2020.01.054 32253097 PMC7306158

[3] Raso MM, Arato V, Gasperini G, Micoli F. Toward a Shigella vaccine: opportunities and challenges to fight an antimicrobial-resistant pathogen. Int. J. Mol. Sci. 2023; 24(5):4649. doi: 10.3390/ijms24054649 36902092 PMC10003550

[4] Kim YJ, Yeo SG, Park JH, Ko HJ. Shigella vaccine development: prospective animal models and current status. Curr. Pharmaceut. Biotechnol. 2013; 14, 903–912. doi: 10.2174/1389201014666131226123900 24372251

[5] Manetu WM, M’masi S, c CW. Diarrhea disease among children under 5 years of age: a global systematic review. 2021.

[6] Giersing BK, Isbrucker R, Kaslow DC, Cavaleri M, Baylor N, Maiga D, et al. Clinical and regulatory development strategies for Shigella vaccines intended for children younger than 5 years in low-income and middle-income countries. The Lancet Global Health. 2023; 11(11):e1819–e1826. doi: 10.1016/S2214-109X(23)00421-7 37858591 PMC10603611

[7] Vubil D, Acácio S, Quintò L, Ballesté-Delpierre C, Nhampossa T, Kotloff K, et al. Clinical features, risk factors, and impact of antibiotic treatment of diarrhea caused by Shigella in children less than 5 years in Manhiça District, rural Mozambique. Infect. Drug Resist. 2018:2095–2106. 30464552 10.2147/IDR.S177579 PMC6219103

[8] Percival SL, Williams DW. Shigella. In: Microbiology of waterborne diseases. edn.: Elsevier; 2014: 223–236.

[9] Muthuirulandi Sethuvel D, Devanga Ragupathi N, Anandan S, Veeraraghavan B: Update on: Shigella new serogroups/serotypes and their antimicrobial resistance. Letters Appl. Microbiol. 2017; 64(1):8–18. doi: 10.1111/lam.12690 27783408

[10] Kahsay AG, Muthupandian S. A review on Sero diversity and antimicrobial resistance patterns of Shigella species in Africa, Asia and South America, 2001–2014. *BMC Res*. *notes*. 2016; 9:1–6. 27576729 10.1186/s13104-016-2236-7 PMC5004314

[11] Zaidi MB, Estrada-García T. Shigella: a highly virulent and elusive pathogen. *Current Tropical Medicine Reports*. 2014; 1:81–87. doi: 10.1007/s40475-014-0019-6 25110633 PMC4126259

[12] Ranjbar R, Farahani A. Shigella: antibiotic-resistance mechanisms and new horizons for treatment. Infect. Drug Resist. 2019:3137–3167. doi: 10.2147/IDR.S219755 31632102 PMC6789722

[13] Nisa I, Haroon M, Driessen A, Nijland J, Rahman H, Yasin N, et al. Antimicrobial resistance of Shigella flexneri in Pakistani pediatric population reveals an increased trend of third-generation cephalosporin resistance. *Current Microbiol*. 2022; 79(4):118. doi: 10.1007/s00284-022-02805-9 35220467

[14] Tickell KD, Brander RL, Atlas HE, Pernica JM, Walson JL, Pavlinac PB. Identification and management of Shigella infection in children with diarrhoea: a systematic review and meta-analysis. The Lancet Global Health. 2017; 5(12):e1235–e1248. doi: 10.1016/S2214-109X(17)30392-3 29132613 PMC5695759

[15] Strockbine NA, Maurelli AT. Shigella. *Bergey’s Manual of Systematics of Archaea and Bacteria*. 2015:1–26.

[16] Sadouki Z, Day MR, Doumith M, Chattaway MA, Dallman TJ, Hopkins KL, et al. Comparison of phenotypic and WGS-derived antimicrobial resistance profiles of Shigella sonnei isolated from cases of diarrhoeal disease in England and Wales, 2015. J. *Antimicrob*. *Chemother*. 2017; 72(9):2496–2502. doi: 10.1093/jac/dkx170 28591819

[17] Rahman MZ, Sultana M, Khan SI, Birkeland N-K. Serological cross-reactivity of environmental isolates of Enterobacter, Escherichia, Stenotrophomonas, and Aerococcus with Shigella spp.-specific antisera. *Current Microbiol*. 2007; 54:63–67. doi: 10.1007/s00284-006-0387-9 17171463

[18] Dhakal R, Wang Q, Lan R, Howard P, Sintchenko V. Novel multiplex PCR assay for identification and subtyping of enteroinvasive Escherichia coli and differentiation from Shigella based on target genes selected by comparative genomics. *J*. *Med*. *microbiol*. 2018; 67(9):1257–1264. doi: 10.1099/jmm.0.000784 29969087

[19] Ferdinand AS, Kelaher M, Lane CR, da Silva AG, Sherry NL, Ballard SA, et al. An implementation science approach to evaluating pathogen whole genome sequencing in public health. *Genome Med*. 2021; 13:1–11. 34321076 10.1186/s13073-021-00934-7 PMC8317677

[20] Atxaerandio-Landa A, Arrieta-Gisasola A, Laorden L, Bikandi J, Garaizar J, Martinez-Malaxetxebarria I, et al. A practical bioinformatics workflow for routine analysis of bacterial WGS data. *Microorganisms*. 2022; 10(12):2364. doi: 10.3390/microorganisms10122364 36557617 PMC9781918

[21] Ayele B, Mekonnen Z, Sisay Tessema T, Adamu E, Tsige E, Beyene G. Antimicrobial Susceptibility Patterns of Shigella Species among Children under Five Years of Age with Diarrhea in Selected Health Centers, Addis Ababa, Ethiopia. *Can*. *J*. *Infect*. *Dis*. *Med*. *Microbiol*. 2023; 2023(1):5379881. doi: 10.1155/2023/5379881 37600752 PMC10435301

[22] Dhiviya Prabaa MS, Naveen Kumar DR, Yesurajan IF, Anandan S, Kamini W, Balaji V. Identification of nonserotypeable Shigella spp. using genome sequencing: a step forward. *Future Sci*. *OA*. 2017; 3(4):FSO229. doi: 10.4155/fsoa-2017-0063 29134117 PMC5674244

[23] Abelman RL M’ikanatha NM, Figler HM, Dudley EG. Use of whole genome sequencing in surveillance for antimicrobial-resistant Shigella sonnei infections acquired from domestic and international sources. *Microb*. *Genomics*. 2019; 5(5):e000270. 10.1099/mgen.0.000270 PMC6562246 31099740

[24] Hosangadi D, Smith PG, Giersing BK. Considerations for using ETEC and Shigella disease burden estimates to guide vaccine development strategy. *Vaccine*. 2019; 37(50):7372–7380. doi: 10.1016/j.vaccine.2017.09.083 29031690 PMC6892262

[25] Dutta S, Jain P, Nandy S, Matsushita S, Yoshida S-i. Molecular characterization of serologically atypical provisional serovars of Shigella isolates from Kolkata, India. *J*. *Med*. *Microbiol*. 2014; 63(12):1696–1703. 25261061 10.1099/jmm.0.081307-0

[26] Connor TR, Barker CR, Baker KS, Weill F-X, Talukder KA, Smith AM, et al. Species-wide whole genome sequencing reveals historical global spread and recent local persistence in Shigella flexneri. Elife. 2015; 4:e07335. doi: 10.7554/eLife.07335 26238191 PMC4522646

[27] Yang C, Li P, Zhang X, Ma Q, Cui X, Li H, et al. Molecular characterization and analysis of high-level multidrug-resistance of Shigella flexneri serotype 4s strains from China. Scientific reports. 2016; 6(1):29124. doi: 10.1038/srep29124 27374009 PMC4931504

[28] Sethuvel DPM, Anandan S, Ragupathi NKD, Gajendiran R, Kuroda M, Shibayama K, et al. IncFII plasmid carrying antimicrobial resistance genes in Shigella flexneri: vehicle for dissemination. *J*. *Global Antimicrob*. *Resist*. 2019; 16:215–219. 10.1016/j.jgar.2018.10.014 30342929

[29] Torrez Lamberti MF, Terán LC, Lopez FE, de las Mercedes Pescaretti M, Delgado MA. Genomic and proteomic characterization of two strains of Shigella flexneri 2 isolated from infants’ stool samples in Argentina. BMC genomics. 2022; 23(1):495. 35804311 10.1186/s12864-022-08711-5 PMC9264714

[30] Sethuvel DPM, Perumalla S, Anandan S, Michael JS, Ragupathi NKD, Gajendran R, et al. Antimicrobial resistance, virulence & plasmid profiles among clinical isolates of Shigella serogroups. Indian J. Med. Res. 2019; 149(2):247–256. 31219090 10.4103/ijmr.IJMR_2077_17 PMC6563743

[31] Lefèvre S, Njamkepo E, Feldman S, Ruckly C, Carle I, Lejay-Collin M, et al. Rapid emergence of extensively drug-resistant Shigella sonnei in France. *Nat*. *Communicat*. 2023; 14(1): 462. doi: 10.1038/s41467-023-36222-8 36709320 PMC9883819

[32] Bunduki GK, Heinz E, Phiri VS, Noah P, Feasey N, Musaya J. Virulence factors and antimicrobial resistance of uropathogenic Escherichia coli (UPEC) isolated from urinary tract infections: a systematic review and meta-analysis. *BMC infect*. *Dis*. 2021; 21:1–13. 34348646 10.1186/s12879-021-06435-7 PMC8336361

[33] Fatima S, Akbar A, Irfan M, Shafee M, Ali A, Ishaq Z, et al. Virulence Factors and Antimicrobial Resistance of Uropathogenic Escherichia coli EQ101 UPEC Isolated from UTI Patient in Quetta, Balochistan, Pakistan. *BioMed Res*. *Int*. 2023; 2023. 10.1155/2023/7278070 PMC10506881 37727279

[34] Tóth AG, Csabai I, Judge MF, Maróti G, Becsei Á, Spisák S, et al. Mobile antimicrobial resistance genes in probiotics. *Antibiotics*. 2021; 10(11):1287. doi: 10.3390/antibiotics10111287 34827225 PMC8614787

[35] Gordienko EN, Kazanov MD, Gelfand MS. Evolution of pan-genomes of Escherichia coli, Shigella spp., and Salmonella enterica. *J*. *Bacteriol*. 2013; 195(12):2786–2792. doi: 10.1128/JB.02285-12 23585535 PMC3697250

[36] King LM, Lovegrove MC, Shehab N, Tsay S, Budnitz DS, Geller AI, et al. Trends in US outpatient antibiotic prescriptions during the coronavirus disease 2019 pandemic. *Clin*. *Infect*. *Dis*. 2021; 73(3):e652–e660. doi: 10.1093/cid/ciaa1896 33373435 PMC7799289

[37] WHO. WHO report on surveillance of antibiotic consumption: 2016–2018 early implementation. 2018. Available online: https://iris.who.int/handle/10665/277359 (accessed on 5 February 2022).

[38] Elkenany R, Eltaysh R, Elsayed M, Abdel-Daim M, Shata R. Characterization of multi-resistant Shigella species isolated from raw cow milk and milk products. *J*. *Vet*. *Med*. *Sci*. 2022; 84(7):890–897. doi: 10.1292/jvms.22-0018 35527016 PMC9353095

[39] Nishino K, Yamaguchi A. Role of histone-like protein H-NS in multidrug resistance of Escherichia coli. *J*. *bacteriol*. 2004; 186(5):1423–1429. doi: 10.1128/JB.186.5.1423-1429.2004 14973023 PMC344412

[40] Anderson M, Sansonetti PJ, Marteyn BS. Shigella diversity and changing landscape: insights for the twenty-first century. *Front*. *Cell*. *Infect*. *Microbiol*. 2016; 6:45. doi: 10.3389/fcimb.2016.00045 27148494 PMC4835486

