# Supplementary material for: Whole genome sequencing and antimicrobial resistance among clinical isolates of Shigella sonnei in Addis Ababa, Ethiopia
Source: PLoS One. 2024 Nov 12;19(11):e0313310. doi: 10.1371/journal.pone.0313310 (PMC11556702; doi:10.1371/journal.pone.0313310)
Supplement: S1 Table — S, Sensitive; I, Intermediate; R, resistant. Notice: any results related to phase one study can be accessible at: Ayele, B.; Mekonnen, Z.; Sisay Tessema, T.; Adamu, E.; Tsige, E.; Beyene, G. Antimicrobial Susceptibility Patterns of Shigella Species among Children under Five Years of Age with Diarrhea in Selected Health Centers, Addis Ababa, Ethiopia. Can. J. Infect. Dis. Med. Microbiol. 2023, 2023(1), 5379881. (DOCX) [file pone.0313310.s001.docx]

| Antimicrobial agents tested | Sensitivity pattern (n=47), n (%) | | |
| --- | --- | --- | --- |
|  | S | I | R |
| Ampicillin | 3(6.4) | 0(0) | 44(93.6) |
| Co-trimoxazole  (trimethoprim) | 11(23.4) | 9(19.2) | 27(57.5) |
| Ciprofloxacin | 38(80.9) | 3(6.4) | 6(12.8) |
| Tetracycline | 4(8.5) | 0(0) | 43(91.5) |
| Doxycycline | 5(10.6) | 3(6.4) | 39(83) |
| Erythromycin | 0(0) | 0(0) | 47(100) |
| Chloramphenicol | 7(14.9) | 8(17) | 32(68.1) |
| Amoxicillin | 0(0) | 0(0) | 47(100) |
| Cefoxitin | 27(57.5) | 10(21.3) | 10(21.3) |
| Gentamicin | 34(72.3) | 3(6.4) | 10(21.3) |
| Norfloxacin | 47(100) | 0(0) | 0(0) |
| Nalidixic acid | 44(93.6) | 0(0) | 3(6.4) |
