# Supplementary material for: Whole genome sequencing and antimicrobial resistance among clinical isolates of Shigella sonnei in Addis Ababa, Ethiopia
Source: PLoS One. 2024 Nov 12;19(11):e0313310. doi: 10.1371/journal.pone.0313310 (PMC11556702; doi:10.1371/journal.pone.0313310)
Supplement: S2 Table — (DOCX) [file pone.0313310.s002.docx]

| Antimicrobial agent | Disk content/ μg | Zone diameter interpretive criteria(nearest whole mm) | | |
| --- | --- | --- | --- | --- |
|  |  | S | I | R |
| Ampicillin | 10 | >17 | 14-16 | <13 |
| Co- trimoxazole | 25 | >16 | 11-15 | <10 |
| Ciprofloxacin | 5 | >26 | 22-25 | <21 |
| Tetracycline | 30 | >15 | 12-14 | <11 |
| Amoxicillin | 10 | >17 | 14-16 | <13 |
| Erythromycin | 15 | >23 | 14-22 | <13 |
| Gentamicin | 10 | >15 | 13-14 | <12 |
| Norfloxacin | 10 | >17 | 13-16 | <12 |
| Nalidixic acid | 30 | >19 | 14-18 | <13 |
| Doxycycline | 30 | >14 | 11-13 | <10 |
| Cefoxitin | 10 | >18 | 15-17 | <14 |
| Chloramphenicol | 30 | >18 | 13-17 | <12 |
